# Accuracy of Self-Reported Screening Mammography Use: Examining Recall among Female Relatives from the Ontario Site of the Breast Cancer Family Registry

**DOI:** 10.1155/2013/810573

**Published:** 2013-08-01

**Authors:** Meghan J. Walker, Anna M. Chiarelli, Lucia Mirea, Gord Glendon, Paul Ritvo, Irene L. Andrulis, Julia A. Knight

**Affiliations:** ^1^Prevention and Cancer Control, Cancer Care Ontario, Toronto, ON, Canada M5G 2L7; ^2^Division of Epidemiology, Dalla Lana School of Public Health, University of Toronto, Toronto, ON, Canada M5T 3M7; ^3^Maternal-Infant Care Research Centre, Mount Sinai Hospital, Toronto, ON, Canada M5G 1X6; ^4^Division of Biostatistics, Dalla Lana School of Public Health, University of Toronto, Toronto, ON, Canada M5T 3M7; ^5^Lunenfeld-Tanenbaum Research Institute, Mount Sinai Hospital, Toronto, ON, Canada M5G 1X5; ^6^School of Kinesiology and Health Science, Faculty of Health, York University, Toronto, ON, Canada M3J 1P3; ^7^Department of Molecular Genetics, University of Toronto, Toronto, ON, Canada M5S 1A8

## Abstract

Evidence of the accuracy of self-reported mammography use among women with familial breast cancer risk is limited. This study examined the accuracy of self-reported screening mammography dates in a cohort of 1,114 female relatives of breast cancer cases, aged 26 to 73 from the Ontario site of the Breast Cancer Family Registry. Self-reported dates were compared to dates abstracted from imaging reports. Associations between inaccurate recall and subject characteristics were assessed using multinomial regression. Almost all women (95.2% at baseline, 98.5% at year 1, 99.8% at year 2) accurately reported their mammogram use within the previous 12 months. Women at low familial risk (OR = 1.77, 95% CI: 1.00–3.13), who reported 1 or fewer annual visits to a health professional (OR = 1.97, 95% CI: 1.15, 3.39), exhibited a lower perceived breast cancer risk (OR = 1.90, 95% CI: 1.15, 3.15), and reported a mammogram date more than 12 months previous (OR = 5.22, 95% CI: 3.10, 8.80), were significantly more likely to inaccurately recall their mammogram date. Women with varying levels of familial risk are accurate reporters of their mammogram use. These results present the first evidence of self-reported mammography recall accuracy among women with varying levels of familial risk.

## 1. Introduction

Having a family history of breast cancer has been established as one of the most important risk factors for the development of breast cancer [1–3]. A reduction in breast cancer mortality attributable to mammography among women aged 50 to 74 has been demonstrated [4–6]. In Canada, average-risk women aged 50 to 74 are recommended to undergo screening mammography every 2 to 3 years [7]. In Ontario, mammography is available to women aged 50 to 74 through the Ontario Breast Screening Program (OBSP), and with physician referral through imaging facilities outside of the screening program [8]. Screening guidelines for high-risk women, based on expert opinion, typically include annual mammography and/or MRI starting at age 40 or 10 years prior to the earliest age of onset observed in the family, or as young as age 25 for BRCA mutation carriers [9–15]. In 2011, the OBSP was expanded to include annual combined MRI and mammography screening for women aged 30 to 69 considered to be at very high risk of breast cancer (i.e., BRCA1/2 mutation carriers or family history suggestive of hereditary breast cancer) (Cancer Care Ontario, Internal Communication, 2011). 

Self-reported data is often used in epidemiologic research evaluating the use of cancer screening. The validity of self-reported mammography use has been studied extensively in women with population-level breast cancer risk [16–18]. Overall, the validity of self-reported data has been demonstrated [17, 18]; however, it is less accurate in determining the precise timing of screening. Women often underestimate the amount of time since their last mammogram [17–23]. This phenomenon, known as “telescoping,” occurs when events are reported as being more recent than when they actually occurred [24]. 

Few studies have validated self-reported mammogram data among women with familial risk [25, 26]. These two studies have only included women with very strong familial breast cancer histories, who likely differ in their breast cancer screening behaviors or recall of these behaviors compared to women in the broader population with familial risk. To the authors' knowledge, the accuracy of screening mammography recall among women with varying levels of familial history has not previously been examined. The objectives of the present study were to examine the accuracy of self-reported screening mammogram dates among women with varying levels of familial breast cancer risk, determine the direction of inaccurate recall, and examine factors associated with inaccurate recall.

## 2. Materials and Methods

### 2.1. Study Population

This study utilized data from a cohort of female relatives of incident cases of invasive breast cancer identified from the Ontario site of the Breast Cancer Family Registry (BCFR) funded by the United States National Cancer Institute. Details of the BCFR and the Ontario site have been previously described [27, 28]. Briefly, cases of pathologically-confirmed invasive breast cancer (probands), diagnosed between 1996 and 1998, were identified from the Ontario Cancer Registry. Physicians were contacted to obtain permission to mail their patients a cancer *Family History Questionnaire* (*FHQ*). Respondents meeting a defined set of genetic risk criteria and a random sample (25%) of those not meeting criteria were asked to participate in the Ontario site of the BCFR. Of the 2,587 eligible women, 1,851 (71.5%) probands participated. Probands were then asked for permission to contact specific living relatives (first-degree, those affected with breast, ovarian or certain other cancers, and their first-degree relatives). An invitation letter to participate in the Ontario site of the BCFR was sent to all relatives (*n* = 8, 416), and the 5,122 who agreed to be contacted were mailed an *Epidemiology Questionnaire* (*EQ*) from 1998 to 2004. 

This prospective cohort study was conducted several years after initial recruitment of relatives to the Ontario site of the BCFR. Baseline, year 1, and year 2 *Personal History and Screening Questionnaires* (*PHSQ*) were administered to update changes in demographic characteristics, cancer screening behaviors and breast outcomes. All female relatives enrolled in the Ontario site of the BCFR, who completed an *EQ* and were 20 to 69 years of age and unaffected by breast cancer at the time of the proband's diagnosis, were eligible to participate. From the 3,374 participating female relatives, 1,514 met all study criteria. A baseline *PHSQ *was sent between 2005 and 2007 to the 1,514 eligible women. Of these women, 1,114 (73.6%) completed the baseline *PHSQ*. A year 1 *PHSQ* was sent to 1,077 eligible women approximately one year following the baseline *PHSQ* and 975 (90.5%) completed it. A year 2 *PHSQ* was sent to 969 eligible women and 882 (91.0%) completed it.

### 2.2. Validation Sample

 Women who completed baseline (*n* = 1114), year 1 (*n* = 975), and year 2 (*n* = 882) *PHSQ*s and reported having a mammogram since their previous interview (*n* = 885 at baseline, *n* = 557 at year 1, *n* = 544 at year 2) were eligible. Women were excluded if they had a personal breast cancer diagnosis, they did not have a first-degree family history of breast or ovarian cancer, they did not provide consent to release the imaging report, the imaging report was not available from the imaging centre, or the indication for the mammogram was for nonscreening purposes. Final sample sizes included 699 women at baseline, 469 at year 1 and 456 at year 2. This study was approved by the Research Ethics Boards of Mount Sinai Hospital, the University Health Network, and University of Toronto. 

### 2.3. Data Collection

Information was obtained from four questionnaires. The first (*EQ*) was self-administered during recruitment of female relatives to the Ontario site of the BCFR and collected detailed information on demographics and key behavioral risk factors for breast and ovarian cancer. As several years had elapsed since completion of the *EQ*, three subsequent questionnaires (baseline, year 1, and year 2 *PHSQ*) of similar contents were telephone-administered to update changes in key demographic and health behavior characteristics and collect detailed information on breast cancer screening. Eligible participants were sent an introductory letter with a copy of the *PHSQ* approximately two weeks prior to being contacted by telephone. This allowed participants to recall dates and events and allowed reference to the questionnaire during the interview. This method was found to result in higher response rates and more complete data than achieved by self-administered questionnaires [29]. The questionnaire instruments have been previously described [28, 30, 31]. 

### 2.4. Data Measures

Self-reported dates and reasons for mammograms were obtained from *PHSQ* interviews. The baseline *PHSQ* asked women if they had a mammogram since *EQ* completion. The year 1 and 2 *PHSQ*s asked women if they had a mammogram since completing the last *PHSQ*. Women were asked to provide the date (month and year) or their age at last mammogram. Women were also asked to indicate if the main reason for the last exam was for screening (part of a regular checkup, the OBSP, or have a family history of breast cancer), or nonscreening (breast problem/symptom, follow-up of a previous breast problem, or participation in a research study). For women who provided consent, imaging reports were obtained from the imaging facility and abstracted for: (i) imaging date (day, month, and year); (ii) indication (screening or diagnostic); and (iii) breast imaging-reporting and data system (BI-RADS) classification [32]. 

Classifications of familial breast cancer risk were based on data collected by the cancer *FHQ* completed by the proband, using a modified definition of previously referenced groups for familial breast cancer risk [10, 33].Table 1 shows the criteria for classifying women as low, moderate, or high familial risk. Age at interview was calculated as the difference in years between date of birth and date of the interview. Descriptive analyses used categories of less than 40 years, 40 to 49 years, 50 years and above, while all models were adjusted for age as a continuous variable. Marital status (married/common law, single), education (high-school or less, some college, university, vocational or technical school, Bachelor's degree, or higher), and frequency of visiting a healthcare professional in the past 2 years (once per year or less, 2 to 3 times per year, 4 or more times per year) were determined using responses from the baseline *PHSQ*. Use of clinical breast examination (CBE) and BRCA1/2 genetic testing was updated at each *PHSQ* and based on self-reported use since the previous interview. Perceived risk of developing breast cancer (much below or below average, same as average, above or much above average) was updated at each *PHSQ* and determined using a question adopted from Lipkus et al. [34]. Women were asked, “compared with other women your age, how likely are you to get breast cancer in your lifetime?” Time since last mammogram was calculated as the difference in days between the date on the imaging report and interview date. Descriptive analyses used categories of within 12 months and more than 12 months ago, while models were adjusted using days since last mammogram.

### 2.5. Statistical Analysis

Distributions of sample characteristics at each *PHSQ* were summarized. Self-reported screening mammogram dates and abstracted imaging dates (“gold standard”) were compared to assess rates of agreement. Inaccurate recall was classified as overestimation or underestimation of the time since last mammogram. The difference in months between self-reported and abstracted mammogram dates was compared among women who inaccurately reported mammogram dates. Multinomial logistic regression was used to estimate adjusted associations between inaccurate recall and a number of sociodemographic, health behavior and cancer screening characteristics. All models included familial breast cancer risk, age at interview, and number of days since last mammogram (except for models including time since last mammogram in categories). As women are clustered within families, robust variance estimation was used to estimate all confidence intervals (CI) [35, 36]. All analyses were conducted using SAS, Version 9.2 (SAS Institute Inc., 2004) and the significance of statistical tests was evaluated using two-sided *P* values at a 5% testing level. 

## 3. Results

At baseline, 42.6% of women were classified as low familial risk, while 26.2% and 31.2% were moderate and high risk, respectively (Table 2). The majority of women were 50 years of age or older (63.0%), while 31.3% were aged 40 to 49 and 5.7% were under the age of 40. Almost all women (97.7%) reported a CBE but had not undergone genetic testing (82.9%) since completion of the *EQ*. Most women (61.5%) perceived their lifetime breast cancer risk to be higher than average. Since completion of the *EQ*, 34.3% reported a mammogram within the previous 6 months, 33.1% reported a mammogram 7 to 12 months prior, and 32.6% reported that their last mammogram was more than 12 months prior. Distributions of sociodemographic and health behavior characteristics were similar at years 1 and 2. 

Most women were able to report the month and year (73.8% at baseline, 88.7% at year 1, and 93.0% at year 2) of their last mammogram, while 24.2% at baseline, 11.1% at year 1 and 6.6% at year 2 were only to report the year or their age (Table 3). Among women who reported the month and year, the majority were accurate, though rates of accuracy were higher at year 1 (78.9%) and year 2 (80.4%) than at baseline (61.8%), as the recall period at baseline (time between completing the *EQ* and *PHSQ*) was longer (5 to 7 years). Among women who inaccurately recalled the date of their last mammogram, proportions of women who underestimated and overestimated this time interval were approximately equal at baseline and year 1, but more women overestimated at year 2. Women who underestimated this interval did so by a longer amount of time at baseline and year 2, compared with women who overestimated (5.48 months versus 3.54 months at baseline, 2.35 months versus 1.56 months at year 2). This difference was statistically significant at baseline (*P* = 0.036). 

Among women who reported the month and year of their last mammogram, most (80.8% at baseline, 94.4% at year 1, and 98.1% at year 2) reported it was within the previous 12 months (Table 4). This was confirmed for almost all women (95.2% at baseline, 98.5% at year 1, and 99.8% at year 2), when self-reported and imaging dates were compared. 

At baseline, women with low familial risk were more likely to overestimate the time since last mammography compared to high-risk women (OR = 1.77; 95% CI: 1.00, 3.13). Women who reported an average of 1 or fewer visits to a health professional per year were almost twice as likely to overestimate the time since last mammogram (OR = 1.97; 95% CI: 1.15, 3.39) compared to women with 2 to 3 visits per year, as were women who perceived their breast cancer risk to be below or the same as average (OR = 1.90; 95% CI: 1.15, 3.15) (Table 5). Women who had mammograms more than 12 months prior were more than five times as likely to underestimate the time since last mammogram (OR = 5.22; 95% CI: 3.10, 8.80). At year 1 and year 2, only time since last mammogram remained statistically significant following adjustment (results not shown).

## 4. Discussion

Our study presents some of the first evidence of the accuracy of self-reported screening mammography use among women with varying levels of familial risk. We found that self-reported screening mammogram use within a 12-month period is highly accurate (over 95%). Additionally, 62% of women at baseline, 79% at year 1, and 80% at year 2 accurately reported the exact timing (month and year) of their last screening mammogram. While we did not find systematic evidence of telescoping, we found that the difference in months between self-reported and abstracted dates was significantly larger in women who telescoped the date at baseline. Fewer visits to a health professional per year, lower perceived breast cancer risk, and having a mammogram more than 12 months prior were significantly associated with inaccurate recall, while the association between inaccurate recall and level of familial risk approached significance. 

The high rates of recall accuracy we observed differ from previous studies, most of which focused on women in the general population. For example, two studies [21, 37] reported that approximately 70% of women who reported undergoing mammography in the past year had actually done so. Lower levels (48%) of recall accuracy for mammogram use in the previous year among low-income minority populations have also been reported [38, 39]. Our finding was not unanticipated, as women with heightened risk are likely to be more conscious of their screening behaviors than women in the general population. Accordingly, Larouche et al. [26], who examined recall among a population of women with high familial breast cancer risk, found corresponding administrative records for 85% of women who reported a mammogram in the 12 months following genetic testing.

Telescoping has been one of the most consistent findings of previous studies [17–23]. Pijpe et al. [25], who examined the validity of self-reported lifetime mammogram histories among BRCA1/2 mutation carriers, found that women underestimated the time since last mammogram. While telescoping was minimal in our study, we did find that women who underestimated the time since their last screening mammogram did so by a significantly longer period of time compared with women who overestimated this interval.

Studies which have previously examined predictors of mammogram recall have reported inconsistent results, likely due to differences in study populations and definitions of accurate recall. When examining predictors of recall, most previous studies have not further classified inaccurate recall as overestimation or underestimation of the time since last mammogram. Pijpe et al. [25], who did, examined recall among BRCA1/2 mutation carriers and similarly found that a longer recall period was associated with greater likelihood of underestimating time since last mammogram. While our results at years 1 and 2 are similar to those observed by Pijpe et al. [25], we also found that the odds of overestimating the time since last mammogram were approximately twofold higher for women who reported fewer visits to a health professional and had a lower perceived breast cancer risk at baseline. Two studies similarly found that women with higher perceived risk more accurately report their mammogram use; however, these relationships were not statistically significant [20, 26]. We found that women with a lower familial risk were more likely to overestimate the time since last mammogram. While no studies have previously evaluated mammogram recall by gradients of risk, two studies have demonstrated that women with a first-degree familial breast cancer history are more likely to accurately recall their mammogram use compared to women without a family history [20, 40].

Our study is unique in that it is one of the first to examine the accuracy of self-reported mammography data among a population of women with a range of family histories of breast cancer. The population-based recruitment, large sample size, longer follow-up period, and exclusion of diagnostic mammograms are also notable strengths. This study has several limitations. Due to the challenge of verifying reports from multiple service providers, this study was not designed to assess recall among women who reported nonuse of mammography. Previous evidence suggests women are unlikely to falsely report the nonuse of mammography [20, 22, 23]. In a small number of cases where women reported a mammogram, imaging centres could not provide a report. As it was not possible to distinguish reports missing because the woman did not have a mammogram, from reports missing because the respondent incorrectly recalled the screening facility, these participants were excluded (*n* = 3 at baseline, *n* = 22 at year 1, *n* = 16 at year 2). As women were mailed a copy of the questionnaire approximately two weeks before being contacted by study staff, they could have checked their personal records or otherwise verified the date of their last mammogram prior to completing the questionnaire. This may have inflated rates of recall accuracy. Finally, it should be noted that the accuracy of recall may differ in populations with different breast cancer screening recommendations and systems of healthcare. 

Self-reported mammography use data is widely relied upon in epidemiologic research for measuring adherence to breast cancer screening guidelines. Overall, our study found women with familial breast cancer risk to be extremely accurate reporters of their mammogram use within 12 months and reasonably reliable reporters of the exact timing of their mammogram attendance. Recall was poorer among women with low familial breast cancer risk and lower perceived breast cancer risk, and when asked to recall screening episodes more than one year prior. Where possible, strategies demonstrated to improve the accuracy of self-reported data should be employed by researchers [16]. Women who cannot recall the timing of their mammograms continue to present a challenge to clinicians with regard to medical management, researchers, and to breast cancer surveillance efforts. Caution must be exercised when relying exclusively on self-reported data for the purposes of medical decision making and reporting of surveillance statistics.

## Figures and Tables

**Table 1:** Classification of familial risk of breast and/or ovarian cancer.

Familial risk group	Family history of breast and/or ovarian cancer
High	≥2 first-degree relatives diagnosed with breast and/or ovarian cancer at any age
	≥1 first-degree relative(s) diagnosed with both breast and ovarian cancer at any age
	≥1 first-degree relative(s) diagnosed with bilateral breast cancer at any age ≥1 first-degree male relative(s) diagnosed with breast cancer at any age
	Personal history of ovarian cancer

Moderate	Self-reported Ashkenazi Jewish background
	1 first-degree relative diagnosed with breast cancer before the age of 40
	1 first-degree relative diagnosed with ovarian cancer at any age
	1 first-degree relative diagnosed with breast cancer after the age of 40 and ≥2 second-degree relatives diagnosed with breast cancer at any age 1 first-degree relative with breast cancer diagnosed after the age of 40 and ≥1 second-degree male relative(s) diagnosed with breast cancer at any age

Low	1 first-degree relative diagnosed with breast cancer after the age of 40

**Table 2:** Distribution of sociodemographic, health behavior, and cancer screening characteristics for female relatives of the Ontario site of the Breast Cancer Family Registry.

Characteristic, n (%)	Interview		
	Baseline	Year 1	Year 2
	n = 699	n = 469	n = 456
Familial breast cancer risk			
Low	298 (42.6)	190 (40.5)	193 (42.3)
Moderate	183 (26.2)	127 (27.1)	123 (27.0)
High	218 (31.2)	152 (32.4)	140 (30.7)
Age at interview			
<40	40 (5.7)	26 (5.6)	24 (5.3)
40–49	219 (31.3)	116 (24.7)	100 (21.9)
≥50	440 (63.0)	327 (69.7)	332 (72.8)
Education			
≤High school	235 (33.7)	161 (34.3)	149 (32.7)
Some college/university/vocational/technical school	273 (39.1)	186 (39.7)	182 (39.9)
≥Bachelor's degree	190 (27.2)	122 (26.0)	125 (27.4)
Marital status			
Married/common law	572 (82.0)	393 (83.8)	383 (84.0)
Single	126 (18.2)	76 (16.2)	73 (16.0)
Visits to health professional			
≤1 time per year	224 (32.6)	145 (31.2)	137 (30.5)
2-3 times per year	291 (42.3)	218 (47.0)	214 (47.7)
≥4 times per year	173 (25.1)	101 (21.8)	98 (21.8)
Clinical breast examination			
Yes	683 (97.7)	414 (88.5)	395 (87.8)
No	16 (2.3)	54 (11.5)	55 (12.2)
BRCA1/2 genetic test			
Yes	112 (17.1)	4 (0.9)	1 (0.2)
No	542 (82.9)	457 (99.1)	454 (99.8)
Perceived risk of breast cancer			
Below/same as average	255 (38.5)	155 (34.7)	184 (41.3)
Higher than average	407 (61.5)	292 (65.3)	261 (58.7)
Months since last mammogram			
0–6	240 (34.3)	247 (52.7)	246 (54.0)
7–12	231 (33.1)	198 (42.2)	204 (44.7)
>12	228 (32.6)	24 (5.1)	6 (1.3)
Mammographic finding			
Normal/benign	582 (83.3)	383 (81.7)	366 (80.3)
Abnormal/incomplete	117 (16.7)	86 (18.3)	90 (19.7)

Subgroups may not add to stated totals due to missing values; valid percentages reported.

**Table 3:** Accuracy of screening mammography date recall among female relatives from the Ontario site of the Breast Cancer Family Registry.

Screening mammography date recall	Interview		
	Baseline	Year 1	Year 2
	*n* = 699	*n* = 469	*n* = 456
Total recall			
Self-reported month and year, *n* (%)	**516 (73.8)**	** 416 (88.7)**	** 424 (93.0)**
Accurately recalled imaging date	319 (61.8)	328 (78.9)	341 (80.4)
Overestimated imaging date	95 (18.4)	43 (10.3)	52 (12.3)
Underestimated imaging date	102 (19.8)	45 (10.8)	31 (7.3)
Months difference (reported versus imaging), mean (SD)			
Overestimated imaging date	3.54 (5.4)	2.07 (2.1)	1.56 (1.6)
Underestimated imaging date	5.48 (7.3)*	2.04 (1.9)	2.35 (2.5)

Partial recall			
Self-reported year only, *n* (%)	** 136 (19.5)**	** 28 (6.0)**	** 25 (5.5)**
Accurately recalled imaging year	81 (59.6)	22 (78.6)	24 (96.0)
Overestimated imaging year	24 (17.6)	2 (7.1)	1 (4.0)
Underestimated imaging year	31 (22.8)	4 (14.3)	0 (0.0)
Self-reported age only, *n* (%)	** 33 (4.7)**	** 24 (5.1)**	** 5 (1.1)**
Accurately reported imaging age	20 (60.6)	20 (83.3)	2 (40.0)
Overestimated imaging age	5 (15.2)	0 (0.0)	1 (20.0)
Telescoped imaging age	8 (24.2)	4 (16.7)	2 (40.0)

No recall			
Could not recall year or age, *n* (%)	** 14 (2.0)**	** 1 (0.2)**	** 2 (0.4)**

*P = 0.036 for women who underestimated versus overestimated the imaging mammogram date.

**Table 4:** Accuracy of self-reported adherence to screening mammography guidelines among female relatives from the Ontario site of the Breast Cancer Family Registry.

	Interview		
	Baseline	Year 1	Year 2
	*n* = 516*	*n* = 414*	*n* = 424*
Self-reported mammogram within 12 months, *n* (%)	417 (80.8)	391 (94.4)	416 (98.1)
Imaging date within 12 months	397 (95.2)	385 (98.5)	415 (99.8)
Imaging date > 12 months ago	20 (4.8)	6 (1.5)	1 (0.2)
Self-reported mammogram > 12 months ago, *n* (%)	99 (19.2)	23 (5.6)	8 (1.9)
Imaging date > 12 months ago	87 (87.9)	15 (65.2)	5 (62.5)
Imaging date within 12 months	12 (12.1)	8 (34.8)	3 (37.5)

*Women who self-reported the month and year of their last screening mammogram.

**Table 5:** Associations between screening mammography date recall and sociodemographic, health behavior, and cancer screening characteristics at baseline for female relatives of the Ontario site of the Breast Cancer Family Registry (*n* = 516^†^).

Characteristic	Screening mammography recall [*n* (%)]			Adjusted OR (95% CI)^‡^	
	Accurate *n* = 317	Overestimated *n* = 95	Underestimated *n* = 102	Overestimated versus Accurate	Underestimated versus Accurate
Familial breast cancer risk					
High	118 (37.0)	25 (26.3)	31 (30.4)	1.00	1.00
Moderate	85 (26.6)	25 (26.3)	23 (22.5)	1.45 (0.77–2.72)	1.17 (0.60–2.27)
Low	116 (36.4)	45 (47.4)	48 (47.1)	1.77 (1.00–3.13)	1.28 (0.73–3.26)
Age at interview					
≥50	224 (70.2)	65 (68.4)	58 (56.9)	1.00	1.00
40–49	78 (24.5)	26 (27.4)	36 (35.3)	0.96 (0.56–1.63)	1.26 (0.70–2.29)
<40	17 (5.3)	4 (4.2)	8 (7.8)	0.49 (0.14–1.70)	0.74 (0.24–2.27)
Education					
≥Bachelor's degree	81 (25.4)	22 (23.2)	27 (26.5)	1.00	1.00
Some college, university or vocational/technical school	129 (40.4)	39 (41.0)	39 (38.2)	1.16 (0.62–2.16)	0.93 (0.48–1.80)
≤High school	109 (34.2)	34 (35.8)	36 (35.3)	1.16 (0.62–2.17)	1.02 (0.53–1.97)
Marital status					
Married/common law	264 (82.8)	77 (81.0)	84 (82.3)	1.00	1.00
Single	55 (17.2)	18 (19.0)	18 (17.7)	1.11 (0.60–2.06)	1.33 (0.66–2.65)
Visits to health professional					
≤1 time per year	92 (29.1)	40 (43.5)	38 (37.6)	1.97 (1.15–3.39)*	1.42 (0.79–2.57)
2-3 times per year	145 (45.9)	32 (34.8)	41 (40.6)	1.00	1.00
≥4 times per year	79 (25.0)	20 (21.7)	22 (21.8)	1.18 (0.62–2.25)	1.01 (0.53–1.96)
Clinical breast examination					
Yes	313 (98.1)	90 (94.7)	99 (97.1)	1.00	1.00
No	6 (1.9)	5 (5.3)	3 (2.9)	3.10 (0.91–10.50)	1.90 (0.39–9.26)
BRCA1/2 genetic test					
Yes	58 (19.5)	13 (14.1)	19 (20.7)	1.00	1.00
No	239 (80.5)	79 (85.9)	73 (79.3)	1.24 (0.62–2.48)	0.78 (0.39–1.57)
Perceived risk of breast cancer					
Above average	192 (64.0)	44 (47.8)	64 (65.3)	1.00	1.00
Below/same as average	108 (36.0)	48 (52.2)	34 (34.7)	1.90 (1.15–3.15)*	1.05 (0.58–1.88)
Time since last mammogram					
0–12 months	271 (84.9)	73 (76.8)	51 (50.0)	1.00	1.00
>12 months	48 (15.1)	22 (23.2)	51 (50.0)	1.67 (0.94–2.99)	5.22 (3.10–8.80)**
Mammographic finding					
Normal/benign	268 (84.0)	86 (90.5)	79 (77.4)	1.00	1.00
Abnormal/incomplete	51 (16.0)	9 (9.5)	23 (22.6)	0.61 (0.28–1.33)	1.80 (0.97–3.34)

*P < 0.05.

**P < 0.0001.

^†^Women who reported the month and year of their last screening mammogram.

^‡^Models adjusted for familial breast cancer risk, age at interview, number of days since last mammogram, and corrected for familial clustering.
